# Delayed auditory feedback simulates features of nonfluent primary progressive aphasia

**DOI:** 10.1016/j.jns.2014.09.039

**Published:** 2014-12-15

**Authors:** Carolina Maruta, Sonya Makhmood, Laura E. Downey, Hannah L. Golden, Phillip D. Fletcher, Pirada Witoonpanich, Jonathan D. Rohrer, Jason D. Warren

**Affiliations:** a Institute of Molecular Medicine and Faculty of Medicine, University of Lisbon, Portugal; b Dementia Research Centre, UCL Institute of Neurology, University College London, London, United Kingdom

**Keywords:** Delayed auditory feedback, Altered auditory feedback, Dementia, Progressive aphasia, Language, Dorsal pathway

## Abstract

The pathophysiology of nonfluent primary progressive aphasia (nfvPPA) remains poorly understood. Here, we compared quantitatively speech parameters in patients with nfvPPA versus healthy older individuals under altered auditory feedback, which has been shown to modulate normal speech output. Patients (n = 15) and healthy volunteers (n = 17) were recorded while reading aloud under delayed auditory feedback [DAF] with latency 0, 50 or 200 ms and under DAF at 200 ms plus 0.5 octave upward pitch shift. DAF in healthy older individuals was associated with reduced speech rate and emergence of speech sound errors, particularly at latency 200 ms. Up to a third of the healthy older group under DAF showed speech slowing and frequency of speech sound errors within the range of the nfvPPA cohort. Our findings suggest that (in addition to any anterior, primary language output disorder) these key features of nfvPPA may reflect distorted speech input signal processing, as simulated by DAF. DAF may constitute a novel candidate pathophysiological model of posterior dorsal cortical language pathway dysfunction in nfvPPA.

## Highlights

• The pathophysiology of nonfluent progressive aphasia (nfvPPA) is poorly understood.
• Delayed auditory feedback (DAF) disrupts speech output in some normal listeners.
• We compared quantitatively speech in nvfPPA with DAF in healthy older individuals.
• Around a third of healthy older individuals under DAF developed features of nvfPPA.
• DAF is a candidate pathophysiological model of dorsal pathway dysfunction in nfvPPA.

## 1 Introduction

During normal speech production, auditory feedback provides sensory information that is used to fine-tune vocal motor output: where access to this feedback is limited (as in the speech of hearing impaired individuals), speech distortions tend to emerge. In experimental settings, synthetically altered auditory feedback (AAF) has been shown to modulate speech output when applied to a speaker's air-conducted voice [21]. Two forms of AAF, namely delayed auditory feedback (DAF; [10]) and frequency altered feedback [37] have been most extensively studied. Individuals with intrinsically normal speech fluency often show loss of fluency, distorted prosody or articulatory errors under AAF [7], whereas AAF has been used therapeutically in stutterers [3], [24]. Functional brain imaging studies have demonstrated a distributed cortical substrate for AAF in bilateral posterior superior temporal and inferior parietal areas that form part of the dorsal cortical stream for processing speech and other sounds [18], [35]. While a number of detailed accounts of dorsal cortical auditory pathway function have been proposed [19], [20], [26], [32], [41], these generally emphasise intimate sensori-motor linkages between speech perception and production. More particularly, perceptual control of speech production may engage a mechanism in the posterior superior temporal plane (STP) that links auditory vocal representations with articulatory gestures via the dorsal language pathway [41].

Progressive non-fluent aphasia (the nonfluent/agrammatic variant of primary progressive aphasia, nfvPPA) is a canonical neurodegenerative syndrome characterised by slow, effortful, hesitant speech marred by errors of grammar and articulation [13], [14], [27]. It is generally considered a disorder of language output programming, though the pathophysiology of nfvPPA is incompletely understood. Neuroanatomically, nvfPPA is linked to damage in peri-Sylvian cortical regions associated with the dorsal language pathway [1], [25], [30]. The speech disturbance in nfvPPA bears certain similarities to that induced in healthy individuals by AAF: in particular, slowing of speech rate, dysprosody and emergence of articulatory errors. Moreover, patients with nfvPPA have additional deficits in processing complex sounds, including prosody, accents, pitch patterns, voices and environmental noises [11], [12], [15], [16], [28], aligning this syndrome with the wider spectrum of progressive aphasia syndromes [38]. This suggests that AAF and nfvPPA might disrupt language network function by at least partly convergent pathophysiological mechanisms, whereby disordered processing of vocal sensory input contributes to impaired speech output via the dorsal language pathway. AAF techniques have been used to assess mechanisms and to rehabilitate dysarthria and dysphasia in stroke, Parkinson's disease and various other neurodegenerative disorders [4], [6], [9], [17], [39] but have not been applied previously in nfvPPA. Here, we compared quantitatively the speech produced by healthy older individuals under AAF and by patients with nfvPPA. We hypothesised that healthy participants under AAF would show slowing of speech rate and emergence of speech sound errors similar to those exhibited by patients with nfvPPA.

## 2 Material and methods

### 2.1 Participants

The healthy participant group (n = 17; nine males, mean age 67 years, range 50–78 years) comprised older native English speakers with no previous history of developmental dysfluency, stuttering or hearing deficits. Patients with nfvPPA (n = 15; 12 males, mean age 77 years, range 66–84 years) were recruited consecutively from a specialist cognitive disorders clinic; all fulfilled current consensus criteria for nfvPPA [13] and general neuropsychological performance profiles corroborated the syndromic diagnosis in all cases [27]. The nfvPPA and healthy participant groups did not differ in gender composition (χ^2^ = 0.467; p = 0.545), however the nfvPPA group was on average significantly older than the healthy participants (Mann–Whitney U = 134.000; p = 0.03).

Ethical approval for the study was obtained from the Local Research Ethics Committee, and all participants gave written informed research consent.

### 2.2 Experimental procedures

The “Grandfather Passage” ([40]; Supplementary Fig. S1) was chosen as a standardised, representative inventory of English phonemes. Three AAF conditions were created using a commercially available software package, Fluency Coach® (http://www.fluencycoach.com/). A short-latency DAF condition was set at 50 ms, corresponding approximately to the minimum delay at which modulation of fluency has been shown in studies of stuttering [22]; a long-latency DAF condition was set at 200 ms, corresponding approximately to the duration of a syllable in conversational spoken English and associated with maximal fluency disruption in previous work [33]; and a combined AAF condition was set at 200 ms plus an upward pitch shift of 0.5 octaves.

The AAF conditions were administered to healthy participants via Sennheiser® (HD265 Linear) headphones at a comfortable listening level (at least 70 dB) in a quiet room. Participants were instructed to read the passage aloud as naturally as possible. Speech samples were recorded as digital wavefiles using Goldwave® software onto a laptop computer with a built-in microphone, for analysis off-line. Before recording commenced, healthy participants were first familiarised with the AAF procedure and set-up. The order of presentation of AAF conditions was randomised between participants, however the baseline (no AAF) condition was always administered last, to reduce any rehearsal effects; participants were blind to condition order.

Speech wavefiles were initially edited manually to remove any extraneous noise sources or pauses. Mean speech rate for each AAF condition in the healthy participant group and for the nfvPPA group was calculated as the mean number of words produced per second, as determined using a customised programme in MATLAB®. The mean total number of errors for each AAF condition in the healthy participant group and for the nfvPPA group was determined from an acoustic analysis of the speech recordings: errors were further subclassified according to whether they were speech sound errors (syllable duplications, omissions or misarticulations), or grammatical errors (errors of morphology or syntax).

### 2.3 Statistical and qualitative analyses

Statistical analyses were performed using SPSSv17®. Multivariate analyses of variance (MANOVAs) were used to assess the effect of group membership (healthy vs nfvPPA) on behavioural performance in each AAF condition. Age, gender and reverse digit span (an index of auditory working memory potentially relevant to monitoring of speech output under AAF) were incorporated as covariates in group comparisons. MANOVAs were also performed to assess the effect of DAF condition (independent variable: baseline, short-latency DAF, long-latency DAF) on behavioural performance of healthy participants (dependent variables: speech rate, total errors, duplications, misarticulations, omissions); post hoc pair-wise comparisons between conditions using Bonferroni's correction were carried out if significant overall correlations were found. For all tests, results were considered statistically significant at a threshold p < 0.05.

In addition, in order to qualitatively assess the confusability of healthy individuals' speech under AAF with speech produced by patients with nfvPPA, speech samples from the nfvPPA group and the healthy group under DAF were classified according to group membership by an experienced cognitive neurologist (PW) blinded to group membership.

## 3 Results

### 3.1 Group data on reading task

**Fig. 1:**
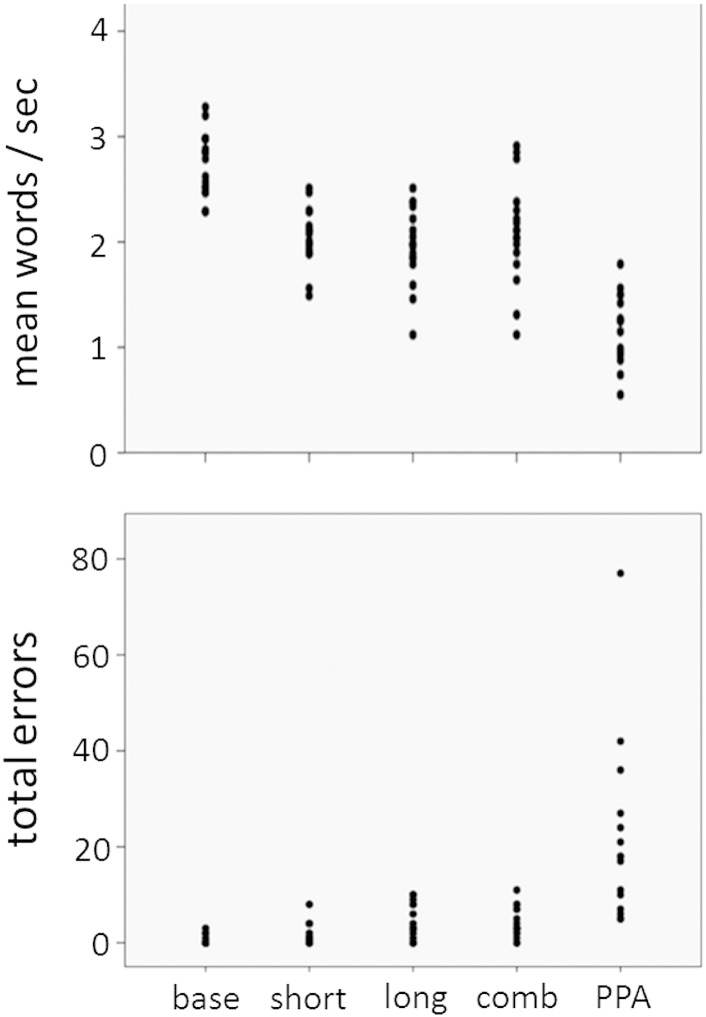
Plots of individual raw scores for mean speech rate and total error scores for healthy older participants under each AAF condition and for patients with nonfluent primary progressive aphasia on reading aloud. The error score is the raw number of errors made over the whole passage. Key: base, healthy individuals baseline (no altered auditory feedback); short, short latency delayed auditory feedback = 50 ms; long, long latency delayed auditory feedback = 200 ms; comb, combined 200 ms delay plus frequency altered (0.5 octave upward) auditory feedback; PPA, nonfluent primary progressive aphasia.

For the reading aloud task, the healthy participant group showed a significantly faster mean speech rate than the nfvPPA group at baseline (F(1,27) = 57.7, p < 0.0001) and this difference remained (but was attenuated) under the short-latency DAF (F(1,27) = 17.9, p < 0.0001), long-latency DAF (F(1,27) = 8.77, p = 0.006) and combined AAF (F(1,27) = 6.34, p = 0.018) conditions. The mean total error score and scores for error subcategories did not differ significantly between the healthy participant and nfvPPA groups at baseline nor under any of the AAF conditions; this was likely attributable to the wide variation in error scores within the nfvPPA group (see Fig. 1). In both the healthy participant and nfvPPA groups, the most frequent speech sound error types were phonemic duplications and misarticulations.

Significant main effects of DAF condition on speech rate (F(2,43) = 29.95, p < 0.0001), total error score (F(2,43) = 10.35, p < 0.0001) and duplication (F(2,43) = 8.05, p = 0.001) and misarticulation (F(2,43) = 6.63, p = 0.003) error scores were found. Speech rate was significantly slower on short-latency and long-latency DAF than on baseline (p < 0.0001). Duplication errors were significantly more frequent in the long-latency DAF condition than at baseline or in the short-latency DAF condition (p < 0.05) and misarticulation errors were significantly more frequent in the long-latency DAF condition than at baseline (p = 0.002).

### 3.2 Individual data: healthy individuals acquiring speech features of nfvPPA under AAF

A proportion of healthy individuals (Fig. 1) showed slowing of mean speech rate and total error rates within the range of patients with nfvPPA. The proportion of healthy participants acquiring these characteristics rose with increasing DAF latency: at a DAF latency of 200 ms, 4/17 (24%) of healthy participants developed a mean speech rate within the nfvPPA range and 6/17 (35%) developed a total error score within the nfvPPA range. Main effects of gender and age on error rates were observed: healthy male participants produced significantly more duplication errors than healthy female participants overall (F(1,43) = 5.88, p = 0.020), and healthy participants made significantly more frequent misarticulation errors with advancing age (F(1,43) = 7.83, p = 0.008).

When speech samples from the nfvPPA group and the healthy participant group under DAF (latency 200 ms) were classified (nfvPPA or healthy) by an experienced cognitive neurologist blinded to group membership, 2/17 (12%) of healthy participant speech samples were misclassified as nfvPPA while all nfvPPA samples were classified correctly.

## 4 Discussion

Here we have shown that AAF, in particular, increasing DAF latency, is associated with significant deterioration in the rate and quality of speech output in healthy older individuals. These findings corroborate previous evidence in younger individuals concerning the effects of DAF latency on speech output [7], [33], [35]. Our data further demonstrate that DAF can induce two cardinal features of nfvPPA, slowing of speech rate and speech sound errors, in a substantial proportion (up to a third) of healthy older individuals. The findings imply that an anterior, primary language output disorder is not essential to produce these key features of nfvPPA — disordered processing of speech input signals (as simulated by DAF) can itself do this.

The question arises as to whether the effects of AAF we have demonstrated were essentially nonspecific and any similarity to nfvPPA therefore purely incidental. We consider this unlikely: in susceptible individuals, the profile of speech sound errors produced was qualitatively as well as quantitatively similar to the profile in nfvPPA, duplications and misarticulations being over-represented in relation to omissions. Moreover, the effects of AAF in healthy individuals here were driven largely by DAF (i.e., manipulation of feedback latency) with little added effect from frequency manipulation. Taken together, this circumstantial evidence argues that DAF was exerting a relatively specific pathophysiological effect and that this effect may have accessed a broadly similar mechanism to the disease process in nfvPPA. The effects of DAF on speech rate and error frequency were strongest at a latency of 200 ms on this reading task. This pattern would be anticipated if DAF principally disrupted the sequential transcoding of phonemes into an ‘automatic’ or obligatory motor speech output: i.e., if DAF acts at the level of the dorsal cortical language pathway [41]. This putative action on the dorsal language pathway would align the DAF paradigm with neuropsychological and structural and functional neuroimaging evidence implicating the dorsal pathway in the pathogenesis of nfvPPA [1], [11], [12], [15], [16], [25], [28], [30].

Accounts of language breakdown in nfvPPA have tended to emphasise the role of anterior brain regions with a primary role in motor speech programming. However, recent work has highlighted more general deficiencies of complex sound analysis in the progressive aphasias that are not primarily motor, or indeed, specifically verbal [11], [12], [15], [16], [28], [38]. This accords both with neuroimaging evidence implicating a distributed brain network and long dorsal white matter tracts in the pathogenesis of nfvPPA [1], [25], [30] and with the concept that the dorsal language and auditory cortical pathways behave as a functional unit with progressive transcoding of information along these pathways [41]. We do not, of course, argue here for a unitary mechanism of nfvPPA: rather, DAF may be modelling a key component of nfvPPA that has been relatively under-recognised, namely, disordered sensori-motor integration that impacts on motor speech output via the dorsal language pathway. In this model, DAF may simply be acting to simulate the effect of ‘noisy’ processing in the dorsal pathway; however, the disease process in nfvPPA might parallel the effects of DAF more closely if, for example, a net reduction of processing speed in damaged cortex disrupts the scheduling of auditory-motor transformations in the dorsal pathway and thereby interferes with feedback controls on speech output [26], [41]. The dynamic nature of DAF may be particularly relevant in an era of increasing interest in pathophysiologically motivated, reversible models of brain damage, notably transcranial magnetic stimulation [36].

The determinants of individual susceptibility to DAF remain largely unknown. In this and in previous studies, age and gender were identified as important modulatory factors [7], [8]. Normal ageing is associated with a generalised slowing of cognitive processing speed [29], which might lead to a correspondingly reduced capacity for tracking alterations of incoming speech signals. This reduction of temporal flexibility might interact with ageing-associated reorganisation of neural networks mediating speech production [31] and executive filtering of auditory inputs [2]. The particular susceptibility of males to DAF may reflect auditory cortical structural and electrophysiological gender differences [5], [34]; these gender effects may modulate auditory-motor integration, and may also contribute to the higher incidence of developmental speech impairments in males [42]. Individual susceptibility factors might be exploited in applying DAF in neurodegenerative disease settings: it might, for example, be feasible particularly in older male individuals to use DAF as a speech output ‘stress test’ in the early stages of progressive aphasia, or to assist in monitoring the impact of therapeutic interventions.

This study should be regarded as preliminary, with several limitations that suggest directions for future work. Larger cohorts are required to substantiate these findings and allow stratification according to specific DAF parameters and individual DAF susceptibility factors, in particular the effects of normal ageing. It will be important to assess the effects of DAF directly in cohorts of patients with progressive aphasia. Future studies should explore the potential of AAF to track the evolution of disease longitudinally across the heterogeneous progressive aphasia spectrum, including the logopenic variant which may be integrally linked to dorsal cortical language pathway dysfunction [14], [25], [27], [36]. The validity of DAF as a pathophysiological model of nfvPPA could be assessed using functional neuroanatomical techniques in parallel cohorts of patients and healthy individuals under DAF: this would help to define the underlying brain mechanism, with the prediction that DAF shifts neural network activity associated with speech production in the healthy brain toward the profile of nfvPPA. It would also be of interest to track adaptation to DAF shown by healthy individuals [23]: the brain mechanisms that support such plasticity might help compensate (or fail to compensate) the effects of brain damage in nfvPPA. We hope that the present data will stimulate further systematic exploration of AAF and related pathophysiological models of progressive aphasia.

The following is the Supplementary data related to this article.

Fig. S1 The grandfather passage (Van Riper, 1963).
