# Supplementary material for: Delayed auditory feedback simulates features of nonfluent primary progressive aphasia
Source: J Neurol Sci. 2014 Dec 15;347(1-2):345–8. doi: 10.1016/j.jns.2014.09.039 (PMC4267508; doi:10.1016/j.jns.2014.09.039)
Supplement: Fig. S1 — The grandfather passage (Van Riper, 1963). [file mmc1.pdf]

SUPPLEMENTARY MATERIAL

Figure S1. The Grandfather Passage (Van Riper, 1963)

You wished to know all about my grandfather. Well, he is nearly ninety-three years old; he dresses himself in an ancient black frock coat, usually minus several buttons; yet he still thinks as swiftly as ever. A long, flowing beard clings to his chin, giving those who observe him a pronounced feeling of utmost respect. When he speaks, his voice is just a bit cracked and quivers a trifle. Twice each day he plays skilfully and with zest upon our small organ. Except in the winter when the ooze or snow or ice prevents, he slowly takes a short walk in the open air each day. We have often urged him to walk more and smoke less, but he always answers, "Banana Oil!" Grandfather likes to be modern in his language.
